# Preparation of Alginate/AgNP Nanocomposite Hydrogels Incorporating Olive Leaf Extracts in Natural Deep Eutectic Solvents

**DOI:** 10.3390/polym17233234

**Published:** 2025-12-04

**Authors:** Ioanna Pitterou, Athina Tzavara Roussi, Aikaterini Malliaraki, Elli Martina Kousouli, Andromachi Tzani, Konstantinos Tsiantas, Anthimia Batrinou, Christina Fountzoula, Anastasios Kriebardis, Panagiotis Zoumpoulakis, Anastasia Detsi

**Affiliations:** 1 Laboratory of Organic Chemistry, Department of Chemical Sciences, School of Chemical Engineering, National Technical University of Athens, 15780 Athens, Greece; 2 Laboratory of Chemistry, Analysis and Design of Food Processes, Department of Food Science and Technology, University of West Attica, 12243 Athens, Greecebatrinou@uniwa.gr (A.B.); pzoump@uniwa.gr (P.Z.); 3 Laboratory of Chemistry, Biochemistry and Cosmetic Science, Department of Biomedical Sciences, School of Health and Caring Sciences, University of West Attica, 12243 Athens, Greece; 4 Laboratory of Reliability and Quality Control in Laboratory Hematology, Department of Biomedical Sciences, School of Health and Caring Sciences, University of West Attica, 12243 Athens, Greece

**Keywords:** silver nanoparticles, nanocomposite hydrogels, NADES, antibacterial activity, green nanotechnology

## Abstract

In the present study, alginate–silver nanoparticle (Alg-AgNP) nanocomposite hydrogels possessing antibacterial activity were synthesized via an innovative route. A task-specific designed Natural Deep Eutectic Solvent (NADES), composed of glucose, lactic acid, and water, was utilized as a green extraction solvent of bioactive compounds from olive leaves (OLs). The NADES–olive leaf extract (NADES-OLE) was used as obtained for the preparation of the Alg-AgNP nanocomposite hydrogel as a multiple-role component. The NADES-OLE acts (a) as a crosslinking agent for the preparation of the alginate hydrogels, (b) as a reducing agent for the in situ synthesis of AgNPs during hydrogel formation, and (c) as a bioactivity enhancement agent due to the presence of compounds obtained from the olive leaves. The Alg-AgNP hydrogel preparation process was optimized through a Box–Behnken experimental design. The resulting nanocomposite hydrogels were characterized for their swelling capacity and water retention in phosphate buffer (pH 5.5), achieving 538% swelling capacity within 180 min and 90% water retention after 250 min. The AgNPs formed within the hydrogels were found to have an average size of 103.2 ± 5.6 nm, with a concentration of 1.2 10^8^ ± 2.2 ∗ 10^7^ particles/mL. Antibacterial testing of the nanocomposite hydrogels against foodborne pathogens, including Gram-negative (*Escherichia coli*, *Salmonella Typhimurium*, *Yersinia enterocolitica*) and Gram-positive bacteria (*Listeria monocytogenes*, *Staphylococcus aureus*, *Bacillus cereus*), revealed significant antibacterial activity, particularly against *E. coli* (64.9%), *Y. enterocolitica* (60.6%), *S. aureus* (79.1%), and *B. cereus* (55.3%), at a concentration of 1 mg/mL.

## 1. Introduction

Silver nanoparticles (AgNPs) exhibit remarkable antibacterial activity [1,2] and have been found to be effective against a broad spectrum of bacteria, fungi, and viruses, making them valuable materials for biomedical applications. The bactericidal effect of silver nanoparticles is believed to be mediated primarily through their interaction with sulfur-containing proteins and amino acids present in bacterial cell membranes. Due to the electrostatic attraction between silver ions and the sulfur groups of these biomolecules, silver binds to them and disrupts essential cellular functions. This process ultimately results in the inactivation and destruction of the entire bacterial cell [3,4]. The most critical factors in AgNPs are size, shape, and surface morphology. The smaller the size, the higher the surface area-to-volume ratio, and thus more molecules are present on the surface and act against bacteria [5].

The controlled release of AgNPs is essential to maximize their effectiveness and minimize their potential toxicity. The incorporation of AgNPs in hydrogels is a new research area, with an increasing number of scientific publications [1,6] paving the way for the development of novel materials. Hydrogels are three-dimensional, crosslinked, and hydrophilic polymer structures that absorb, swell, and retain amounts of water or aqueous fluids [7]. They can be considered very promising biocompatible matrices that can control the release of silver ions from the surface of silver nanoparticles, enhancing wound healing and minimizing bacterial resistance. Among various applications, hydrogels are excellent candidates for wound healing, as they provide a moist microenvironment that promotes re-epithelialization and angiogenesis [8]

The incorporation of AgNPs in alginate hydrogels enhances their antibacterial activity and also contributes to their mechanical strength and stability, making them more effective in various biomedical applications [9,10]. Usually, the incorporation of AgNPs in hydrogels occurs by direct mixing of the AgNP suspension and the polymer solution. The AgNPs are usually synthesized starting from a silver precursor (such as silver nitrate) using a reducing agent. Lately, the synthesis of AgNPs using plant extracts has gained significant attention due to its eco-friendly and cost-effective nature. Plant extracts contain a variety of bioactive compounds such as flavonoids, terpenoids, phenols, alkaloids, and polysaccharides that can act as effective reducing agents [11]. When employing plant extracts for the reduction of a silver precursor, a greener synthesis of AgNPs can be achieved simply by mixing the extract with the aqueous solution of the metal salt at room temperature [12].

In the context of Green Chemistry, new solvents, such as Ionic Liquids (ILs) [13] and Deep Eutectic Solvents (DESs) [14], are gradually being used for AgNP synthesis. DESs are a combination of at least two compounds, a hydrogen bond acceptor (HBA) and a hydrogen bond donor (HBD). The components are mixed in appropriate molar ratios and at a specific temperature, and the interactions between them cause a dramatic reduction in the overall melting point, making it much lower than that of the individual components. Natural Deep Eutectic Solvents (NADESs) are a specific category of DESs that are composed of naturally occurring compounds and thus can be biocompatible. NADES have emerged as suitable solvents for extracting bioactive compounds [15,16,17] and they have been proven to stabilize and “protect” extracted bioactive compounds [18].

In the present study, we chose to use NADESs as extraction solvents of valuable phytochemicals from olive leaves and then use the NADES extracts as reducing agents for AgNP formation. Olive leaves (OLs) were selected as a rich source of polyphenols and other phytochemicals that can act as reducing agents. This selection aligns with the principle of circular economy since an agricultural waste is repurposed for the production of other sustainable products. OL extracts (derived from conventional extractions using water, ethanol–water systems, etc.) have been reported for AgNP preparation [19,20,21]. However, in this study, we choose to develop and use an NADES extract since the NADES offers higher extract stability over time and protects bioactive compounds from external factors. Furthermore, NADES itself can also contribute to the hydrogel formation process as a crosslinking agent. Thus, this approach provides multiple advantages in both nanoparticle synthesis and hydrogel preparation.

Each of the main components of the hydrogel composition—the monomer, the initiator, and the crosslinker—influences its final properties, with the crosslinker being the most crucial component affecting the mechanical strength of the hydrogel [22]. In this study, we used sodium alginate as the main component of the hydrogels because it can also act as an effective reducing and stabilizing agent for silver nanoparticles. Alginate is a natural anionic polysaccharide found in marine algae, consisting of β-D-mannuronic acid (M) and α-L-glucuronic acid (G) linked by 1–4 bonds to form the alginate structure [23]. Due to its hydrogel-forming ability, it is often used as a stabilizer, a thickener, a gelling agent, and an emulsifier in various biomedical applications, such as wound dressing, tissue engineering, and drug delivery [24]. Furthermore, alginate is a highly absorbent, biodegradable material with a reducing effect, and it is known to be effective as a wound healing dressing [25].

The aim of this study is to develop and optimize the process for the synthesis of nanocomposite alginate hydrogels containing AgNPs using an NADES extract from olive leaves as a new sustainable approach. The NADES was task-specifically selected to comprise biocompatible compounds such as glucose, D,L-lactic acid, and water, as well as in order to possess suitable physicochemical properties for the specific application.

## 2. Materials and Methods

### 2.1. Materials

Sodium alginate ((C_6_H_7_O_6_Na)n) was purchased from Glentham Life Sciences (Corsham, Wiltshire, UK). Silver nitrate (AgNO_3_, 99.98%) was obtained from D.F. Goldsmith Chemical & Metal Corporation (Evanston, IL, USA) and D,L-lactic acid (C_3_H_6_O_3_, >80%) from LabKem (Barcelona, Spain). D-(+)-Glucose (C_6_H_12_O_6_) was obtained from Sigma Aldrich Chemical Reagent Co., Ltd. (St. Louis, MO, USA). Potassium dihydrogen phosphate (KH_2_PO_4_) and di- potassium hydrogen phosphate anhydrous A.G. (Κ_2_HPO_4_) were from Penta Chemicals Unlimited (Prague, Czech Republic). The olive leaves used in this study were collected from Kalamon olive trees in the Agrinio region of Aetolia-Acarnania, Greece. Ultrapure water (18.2 MΩ at 25 °C) was used throughout the experiments. All glassware was cleaned with piranha solution. All chemicals and solvents were of analytical grade and were used without further purification.

### 2.2. NADES (Glu:LA:W) Preparation

The NADES, consisting of D-(+)-Glucose, D,L-Lactic acid, and water (Glu:LA:W) in 1:5:6.2 molar ratio was prepared using the heating and stirring method as described in our previous work [17].

### 2.3. Extraction Process

The prepared NADES was used as extraction solvent for the extraction of bioactive compounds from olive leaves. The Ultrasound-Assisted Extraction was conducted using a Sonics VC 400 High-Intensity Processor (Sonics & Materials, Newtown, CT, USA) with a piezoelectric converter and a 6 mm diameter titanium alloy (Ti-6Al-4V) probe. Powdered olive leaves (average size of 2 mm) were suspended in a Glu:LA:W (1:5:6.2) NADES in a ratio of 30:1 *w*/*w*. The mixture was ultrasonicated for 15 min at 120 W using a pulse of 9 s on–3 s off, while being immersed in an ice bath to avoid possible overheating. Then, the mixture was centrifuged at 7000 rpm for 10 min, and the supernatant was filtered under vacuum. This centrifugation step was necessary to remove aggregates and unreacted solids, thereby ensuring a uniform colloidal suspension suitable for the preparation of the nanocomposite hydrogel.

The total phenolic content (TPC) of the olive leaf extract (NADES–OLE) was determined using the Folin–Ciocalteu method as described in our previous studies, with slight modifications [16]. Briefly, stock solutions were prepared by diluting the olive leaf extract to 30% (*v*/*v*) with Milli-Q water, while the chamomile extract was used undiluted. Then, 20 μL of each stock solution was mixed with 1 mL of Milli-Q water, followed by the addition of 100 μL Folin–Ciocalteu reagent and incubation in the dark for 5 min. Subsequently, 300 μL of saturated Na_2_CO_3_ and 580 μL of Milli-Q water were added, and the samples were vortexed and incubated for 1 h in the dark. Absorbance was recorded at 755 nm using a UV–Vis spectrophotometer. Gallic acid was used as the reference standard, and the TPC values were expressed as mg gallic acid equivalents (mg GAE) per gram of dry weight (dw). The TPC of the NADES–OLE was found to be 31.50 ± 1.03 mg GAE/g dw olive leaves.

Total flavonoid content (TFC) was determined using the aluminum chloride colorimetric method with slight modifications [16]. Briefly, stock solutions of the extracts were prepared by diluting the olive leaf extract to 30% (*v*/*v*) with ultrapure water, while the chamomile extract was used undiluted. Then, 50 μL of each stock solution was mixed with 30 μL of 5% sodium nitrite (NaNO_2_) solution in a 1.5 mL Eppendorf tube and allowed to react for 6 min. Subsequently, 60 μL of aluminum chloride (AlCl_3_) solution was added, and the samples were vortexed and incubated for 5 min. Afterwards, 300 μL of 1 M sodium hydroxide (NaOH) and 560 μL of distilled water were added, the mixture was vortexed, and the samples were incubated for 15 min in the dark at room temperature. Catechin (1 mg/mL) was used as the reference standard, and absorbance was measured at 510 nm using a UV–Vis spectrophotometer. The experiments were performed in triplicate, and the TFC of the olive leaf extract was found to be 11.93 ± 0.25 mg CAT/g dw olive leaves.

### 2.4. In Situ Preparation of Nanocomposite Hydrogels (Alg-AgNPs)

**Figure 1:**
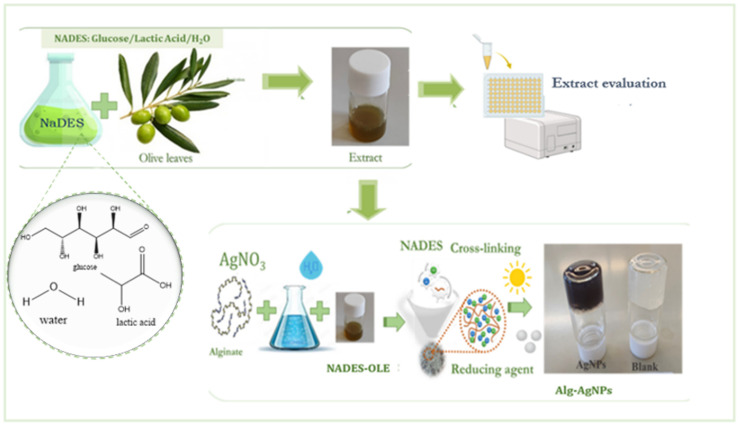
Schematic representation of nanocomposite hydrogel formation.

The one-pot synthesis of the innovative nanocomposite hydrogel is based on the simultaneous formation of silver nanoparticles (AgNPs) and the crosslinking of the polymeric network. The hydrogel samples were prepared using sodium alginate as the polymeric matrix and as a mild reducing agent, silver nitrate as the silver precursor, and the NADES–olive leaf extract (NADES–OLE) as the primary reducing agent and crosslinker (Figure 1). In this system, the contributions of each component to the reduction process are as follows: (a) sodium alginate stabilizes the nanoparticles and provides mild reduction; (b) NADES–OLE acts as the main reducing agent and crosslinker; and (c) sunlight accelerates reduction through photochemical activation.

**Figure 2:**
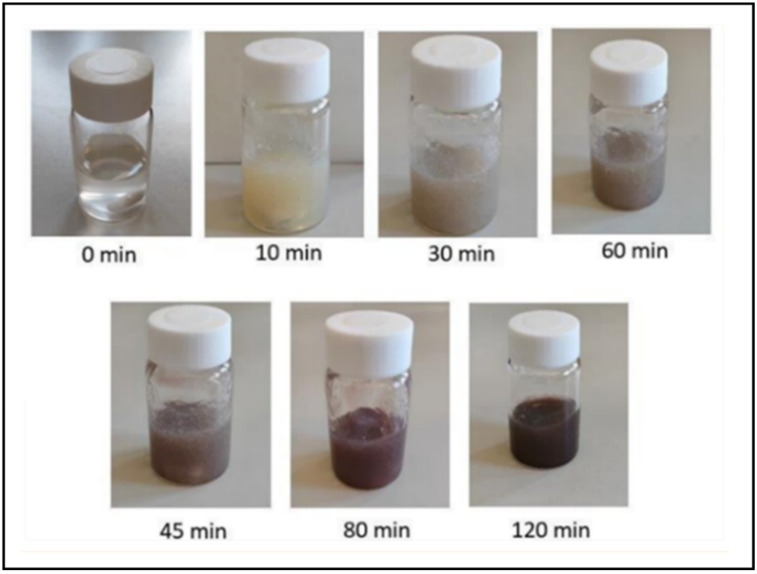
Color change in the nanocomposite hydrogels prepared using NADES-OLE over a period of 2 h.

A 10 mL aliquot of 3% (*w*/*v*) aqueous sodium alginate solution and 550 μL of a 100 mM silver nitrate aqueous solution were added to a glass vial, resulting in a final silver nitrate concentration of 5 mM. Next, 440 μL of NADES-OLE (4% *v*/*v*) was added, and the mixture was stirred under sunlight for 4.5 h at 900 rpm and 25 °C. The nanocomposite hydrogels progressively change color, resulting after 120 min in a dark purple/brown, indicating the formation of silver nanoparticles (AgNPs) (Figure 2).

**Figure 3:**
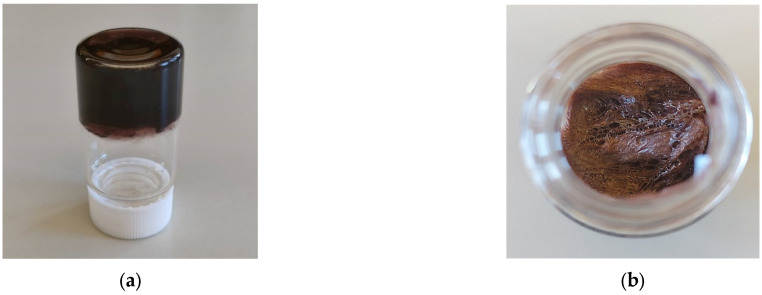
Typical appearance of (**a**) hydrogel before freeze-drying and (**b**) cryogel after freeze-drying.

After gel formation, the samples were frozen and subsequently lyophilized using a Frozen in Time Ltd. Mini Lablyo Freeze Dryer (Model 46/1, York, UK) at a condenser temperature of −50 °C and a chamber pressure of 4 Pa, for 48 h. (Figure 3).

Alginate hydrogels containing either NADES or NADES-OLE were prepared using the same general procedure as the nanocomposite hydrogels, without adding silver nitrate, and the NADES or the NADES-OLE (4% *v*/*v*) was added accordingly.

### 2.5. Optimization of the Nanocomposite Hydrogel Preparation Using Box–Behnken Experimental Design

Response Surface Methodology (RSM) was employed to examine the effect of independent variables on the final product behavior and to predict the optimal conditions for the preparation of the silver nanocomposite hydrogels for their wound healing application. A 15-run Box–Behnken Design (BBD) was established using Design-Expert 12.0 software (trial version), incorporating 3 center points to account for 3 independent factors. Each independent factor is examined at three levels, symbolized as −1 (low), 0 (medium), and +1 (high). The concentration of silver nitrate (mM), the NADES-extract content (% *v*/*v*) and the stirring time (h) were selected as the three independent variables to investigate their effect on two responses: the swelling ratio (%) and the water retention ratio (%) of the nanocomposite hydrogels (Table 1). The statistical significance, which was set at *p* < 0.05, is checked using the analysis of variance (ANOVA).

### 2.6. Nanocomposite Hydrogel Characterization

#### 2.6.1. Swelling Ratio (SR), Water Retention Ratio (WRR)

The swelling capacity of hydrogels was investigated in a phosphate-buffered saline (PBS) solution at pH 5.5 and a temperature of 37 °C, simulating the conditions on the surface of the skin. It was determined by measuring the mass of the swollen hydrogels at specific time intervals [16]. Before weighing, the excess surface water was carefully removed using filter paper. The swelling capacity was then calculated according to Equation (1):

(1) $$\mathrm{Water}\;\mathrm{swelling}\;(\%)\;=\;\frac{m1-\;m0}{m0}*100$$

where m_0_ is the initial weight of the dry sample (cryogel) and m_1_ is the mass swollen weight of the hydrogel at time t.

After the hydrogel reached its maximum swelling capacity (equilibrium) at pH = 5.5, 37 °C, it was used in the following water retention experiments. When the swelling capacity (%) is the maximum, we assume that the water retention (%) of the hydrogel is 100%. Thus, we calculate the water retention with the aid of the following Equation (2):

(2) $$\mathrm{Water}\;\mathrm{retention}\;(\%)\;=\;\frac{m1-\;m0}{m2-m0}*100$$

where m_0_ is the initial weight of the dry sample (cryogel), m_1_ is the mass swollen weight of the hydrogel at time t, and m*_2_* is the weight of the hydrogel at the time of maximum swelling capacity (equilibrium).

#### 2.6.2. UV–Visible Spectroscopy

The UV-Vis spectra of the samples were recorded using a Double-Beam V-770 UV–Vis/NIR Jasco apparatus (Tokyo, Japan) over the range of 300 to 500 nm. For the measurement, a small amount of the nanocomposite hydrogel (1 mg/mL) was diluted in ultrapure water and was left at room temperature for a period of a week.

#### 2.6.3. Nanoparticle Tracking Analysis (ΝΤA)

A Malvern Panalytical NanoSight NS300 (Malvern, UK) was employed to analyze the size distribution and the concentration of the AgNPs. The sample preparation consisted of diluting 10 mg of freeze-dried nanocomposite hydrogel in 10 mL ultrapure water for 2 days. For the measurement, an aliquot of the supernatant containing the AgNPs released from the polymer network was used. 

#### 2.6.4. Zeta Potential Measurements via Electrophoretic Light Scattering (ELS)

The zeta potential, as an index of the surface potential of the AgNPs, was measured using a Malvern Zetasizer Nano ZS (Malvern, UK) at room temperature. Before the measurement, the samples were dissolved in ultrapure water and sonicated for several hours. This treatment contributed to the relaxation of the polymer chains within the hydrogel matrix and the release of the entrapped nanoparticles. A folded capillary cell DTS1070 was used for the assays.

#### 2.6.5. Transmission Electron Microscopy (TEM)

Nanoscale investigation was performed with a high-resolution JEOL JEM-2100 LaB6 transmission electron microscope (HRTEM), operating at 200 kV. The samples under investigation were suspended in deionized water and treated with ultrasound to disaggregate the agglomerated particles. A drop from the suspension was then placed on a 300-mesh carbon-coated copper grid and air-dried overnight.

#### 2.6.6. Antibacterial Activity Assay

Antibacterial activity was tested against five major pathogenic bacteria: the Gram-negative *Escherichia coli* ATCC 25922 and *Yersinia enterocolitica* NCTC12982/ATCC 9610 and the Gram-positive *Listeria monocytogenes* ATCC 35152, *Staphylococcus aureus* ATCC 6538, and *Bacillus cereus* NCTC 10320/ATCC 9634. In this study, hydrogels were dispersed in aqueous medium in a ratio of 1:1 (1 mg/mL). Bacterial strains were incubated at 37 °C overnight in Brain Heart Infusion (BHI) broth and, after vortexing, 100 μL of inoculum was added to a sterilized tube containing 9 mL BHI broth, obtaining a turbidity of 0.5 McFarland standard, which corresponds approximately to 1.5·10^8^ bacteria/mL. Antibacterial activity was tested by automatic turbidometry with Bioscreen C. Samples were loaded in 100-well honeycomb plates by adding 50 μL of the tested hydrogels and controls, 50 μL of microorganism, and 250 μL broth (Brain Heart Infusion, BHI) at a total volume of 350 μL. Conditions were set to measure optical density (OD) of wells at 600 nm, every 30 min for 48 h at 37 °C. Before each measurement, the wells were shaken for 10 s, while all measurements were performed in triplicate. After the data extraction, corresponding graphs of optical density versus time were constructed, which represent the growth curves of microorganisms at the specific conditions. Finally, the results were processed with the ComBase tool DMFit for Excel.

## 3. Results

In the present work, AgNPs were synthesized using a green methodology and were incorporated into alginate hydrogels. A task-specific Natural Deep Eutectic Solvent (NADES) consisting of glucose and lactic acid was used as a solvent to extract bioactive phytochemicals from olive leaves. The as-obtained extract served a dual purpose: (a) as a crosslinking agent for the preparation of alginate hydrogels and (b) as a reducing agent for the synthesis of AgNPs. This environmentally friendly, non-thermal approach was further enhanced by utilizing sunlight as a sustainable energy source to facilitate the reduction process.

### 3.1. Synthesis of Nanocomposite Hydrogels

The incorporation of silver nanoparticles (AgNPs) in hydrogels is crucial for several reasons: (a) AgNPs possess strong antibacterial properties, making the hydrogels effective in preventing infections, especially in wound dressings and other biomedical applications; (b) AgNPs can enhance the mechanical strength and stability of the hydrogels, improving their performance in various applications; (c) AgNPs contribute to the conductive properties of the hydrogels, which is beneficial for applications in biosensors and electronic devices.

A “task-specific” selection of the components used for the NADES was undertaken, keeping in mind that the desired properties of the solvent should be (a) extraction ability in order to extract valuable phytochemicals from plant material, (b) reducing ability to promote the formation of AgNPs, and (c) the ability to act as crosslinking agents to promote the formation of alginate hydrogels. Thus, glucose and lactic acid were chosen as the NADES components (a) because both are biocompatible and safe to use for applications addressing human needs and (b) because glucose is a reducing sugar and can contribute to the formation of AgNPs so that there is no need to add another reducing agent. The glucose–lactic acid–H_2_O NADES is an NADES that we have successfully used in our previous works, and it possesses the characteristics that are favorable for olive leaf extraction: it is a polar solvent (more polar than water), and it has been proven that it can extract phytochemicals more efficiently than the conventional ethanol–water system [15,26].

Ultrasound-Assisted Extraction (UAE) was selected because it provides higher extraction efficiency with reduced processing time and solvent use compared to conventional heating. Beaudor et al. (2023) in their study regarding the UAE of polyphenols from spent coffee grounds showed that ultrasound application allowed about 33% enhancement of polyphenol recovery and more than 50% energy savings compared to conventional extraction [27]. Similarly, Bouaoudia-Madi et al. (2019) reported that UAE of *Myrtus communis* pericarp (70% ethanol, 28 mL/g, 7.5 min) yielded 241.7 ± 12.8 mg GAE/g DW while conventional extraction (50% ethanol, 50 mL/g, 7200 min) resulted in a lower total phenolic content (76.40 ± 7.27 mg GAE/g DW) [28]. The authors noted that UAE provided overall higher total phenolic content of the extract, substantially shorter extraction times, and reduced solvent usage.

The synthesis of the AgNPs inside the polymer network occurred in the presence of sunlight. The photo-induced reduction of Ag^+^ to Ag^0^ using an aqueous extract of chamomile has been recently reported by Alshehri and Malik, who extensively studied the role of sunlight in AgNP formation [11].

### 3.2. UV-Vis Spectroscopy

**Figure 4:**
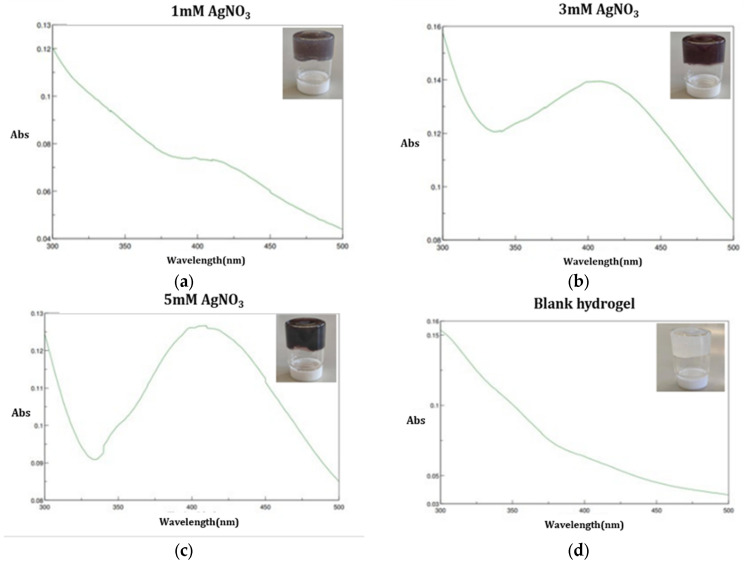
UV-Vis absorption spectrum of the AgNPs released from the nanocomposite hydrogels with a final AgNO_3_ concentration of (**a**) 1 mM, (**b**) 3 mM, and (**c**) 5 mM and (**d**) the spectrum of the blank hydrogel with 0 mM AgNO_3_.

The formation of AgNPs was confirmed by UV–Vis spectroscopy. Figure 4 shows the UV-Vis absorption spectra of the AgNPs released from the nanocomposite hydrogel prepared by NADES-OLE. The spectra in Figure 4a–c exhibit an absorption peak at 420 nm, which is the typical surface plasmon resonance band for silver nanoparticles [13]. The blank hydrogel’s UV–Vis spectrum, as shown in Figure 4d, does not display any absorption band, as it does not contain silver nanoparticles. In contrast, the samples prepared with increasing concentrations of silver nitrate exhibit a progressively more defined and intense absorption peak, reflecting the formation of a greater number of AgNPs. Sivalingam and Pandian similarly reported that higher precursor concentrations lead to sharper and more intense SPR bands, confirming that increased availability of Ag^+^ ions promotes more extensive nanoparticle formation [29]. Furthermore, this characteristic single absorbance at 410–450 nm can be attributed to the formation of spherical nanoparticles.

### 3.3. Experimental Design

In order to optimize the preparation process of the nanocomposite hydrogels, an Experimental Design was undertaken. The factors’ optimal values were determined through experimental design and statistical analysis to maximize the nanocomposite hydrogels’ swelling and water retention ratio. For every response, two equations were generated: the actual and the coded equation. The first equation, which uses actual units, can predict the response value, whereas the second one, which employs coded units and compares coefficients, can determine the relative impact of the factors. ANOVA was used to check the fitness and significance of the model coefficients.

These two responses for each extract were expressed in percentages and are presented in Table 2.

#### 3.3.1. Swelling Ratio of the Nanocomposite Hydrogels

The swelling ratio of the nanocomposite hydrogels after 10 min of immersion in the phosphate-buffered saline (PBS) solution at pH 5.5 and 37 °C was statistically analyzed to determine the effect of the three independent factors. The swelling ratio was best described by the following equations (actual Equation (3) and coded Equation (4), respectively):

(3) $$\%\mathrm{Swelling}\;=\;+2856.05\;-\;8.79\;*\;[{\mathrm{AgNO}}_{3}]\;-\;1258.97\;*\;[\mathrm{NADES}-\mathrm{OLE}]\;+\;\;356.32\;*\;\mathrm{Time}\;+\;6.92\;*\;[{\mathrm{AgNO}}_{3}]\;*\;[\mathrm{NADES}-\mathrm{OLE}]\;+\;10.11\;*\;[{\mathrm{AgNO}}_{3}]\;*\;\;\mathrm{Time}\;-\;87.28\;*\;[\mathrm{NADES}-\mathrm{OLE}]\;*\;\mathrm{Time}\;-\;11.71\;*\;[{\mathrm{AgNO}}_{3}{]}^{2}\;+\;146.05\;*\;\;{[NADES-OLE]\;}^{2}+\;12.26\;*\;{Time}^{2}$$

R_1_ = 181.49 − 7.98 A − 126.88 B + 96.60 C + 13.85 AB + 40.42 AC − 174.57 BC − 46.83 A^2^ + 146.05 B^2^ + 49.04 C^2^ (4)

The proposed quadratic model was significant with the F-value equal to 4.9 and the *p*-value equal to 0.0473 (less than 0.05). The coefficient R^2^ was 0.8983, indicating accuracy and a good fit of the experimental data to the ones predicted from the model, and the adjusted coefficient R^2^ was 0.7152, which verifies the adequacy of the model.

Moreover, the analysis predicted a 9.12 signal-to-noise ratio, which is considered adequate and affirms that the model can be used to navigate the design space. The statistical significance of each model term is shown in Table 3.

The results in Table 4 and in the coded Equation (3) show that the extract content and the stirring time are the most influential factors in the swelling ability of the hydrogel at the first 10 min of immersion.

**Figure 5:**
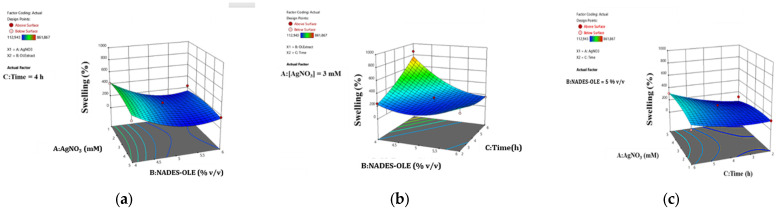
RSM plots for swelling response showing the correlation between swelling ratio and the factors (**a**) NADES-OLE and AgNO_3_, (**b**), time and NADES-OLE, and (**c**) time and AgNO_3_.

The surface response plots in Figure 5 depict the correlation between the studied parameters and the swelling ratio of the nanocomposite hydrogels.

As shown in Figure 5, the nanocomposite hydrogels reach a higher swelling ratio when the extract content is 4% *v*/*v* and the time is 6 h. This observation agrees with the literature, since the NADES responsible for the crosslinking, when used in higher proportions, leadto extensive crosslinking and thus the formation of a tighter network structure, which limits the porosity and therefore the ability to uptake more water molecules [10,30]. From the surface plots in Figure 5, it can be deduced that shorter mixing times result in lower swelling ratios. Since mixing facilitates uniform crosslinking, short stirring times during hydrogel preparation do not allow an increased entanglement and alignment of the polymer chains, restricting the homogeneous distribution of pores and the formation of a finer network structure, which subsequently leads to lower water absorption. Moreover, longer preparation times promote the formation and uniform distribution of more AgNPs, which due to their charged surface contribute to the creation of void spaces within the hydrogel network capable of withholding water. This effect of AgNPs on the network structure and swelling behavior has been previously observed by Porter et al. and Solaiman et al., who reported that AgNPs participate in cross-bridging interactions and reinforce the polymer network, resulting in a stronger and more stable hydrogel structure [9,31].

#### 3.3.2. Water Retention Ratio

For the water retention behavior after 5 h of immersion in the buffer solution, a reduced cubic model can best describe how the factors affect this response. The statistically significant model includes the actual form of Equation (5) as well as the coded Equation (6) and presents a *p*-value of 0.0015 and F-value of 16.43.

(5) $$\%\mathrm{Water}\;\mathrm{Retention}=-648.09+179.50\;*\;[{\mathrm{AgNO}}_{3}]+308.95\;*\;[\mathrm{NADES}-\mathrm{OLE}]\;-27.45\;*\;\mathrm{Time}-70.25\;*\;[{\mathrm{AgNO}}_{3}]\;*\;[\mathrm{NADES}-\mathrm{OLE}]+7.86\;*\;[\mathrm{NADES}-\;\mathrm{OLE}]\;*\;\mathrm{Time}-32.31\;*\;{[NADES-OLE]\;}^{2}-2.26\;*\;{Time}^{2}+6.75\;*\;\;[{\mathrm{AgNO}}_{3}]\;*\;{\;[NADES-OLE]\;}^{2}$$

*R^2^ = 91.46 − 6.00 A + 9.12 B* − *12.38 C − 5.50 AB + 15.75 BC* − *12.06 B*^2^ − *9.06 C*^2^* + 13.50 AB^2^* (6)

This model presented excellent *R*^2^ and *R*_adj_^2^, of 0.9563 and 0.8981, respectively, which indicates that the experimental data are a perfect fit for the model predicted values. Furthermore, the signal-to-noise ratio of 15.52 allows the use of the model to navigate the design space. The statistical significance of each model term is shown in Table 4.

As for the water retention ratio of the hydrogel after 5 h of immersion in the buffer solution, the mixing time and NADES-OLE content are the most statistically significant factors, as is indicated by the *p*-values. The surface response plots depict the correlation between the studied parameters and the water retention ratio of the nanocomposite hydrogels.

**Figure 6:**
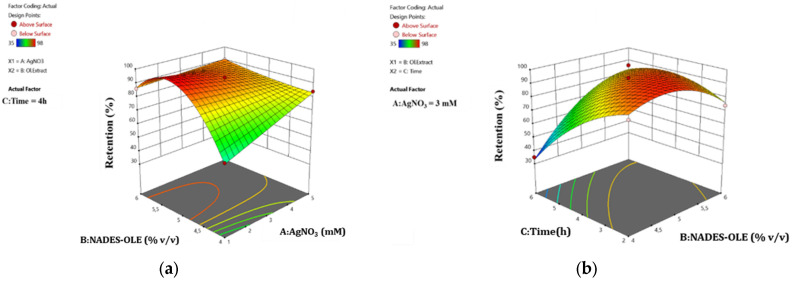
RSM plots for water retention response showing the correlation between water retention ratio and the factors (**a**) NADES-OLE and AgNO_3_ and (**b**) time and NADES-OLE.

As shown in Figure 6, the use of 4% *v*/*v* NADES-OLE (lower limit of experimental design) leads to lower water retention ratios at 300 min of immersion, especially when the silver nitrate concentration is 1 mM (lower limit of experimental design) and time is 6 h (higher limit of experimental design). A higher extract content generally increases the water retention ratio. This can be attributed to the NADES’s crosslinking ability and nature. When the amount of extract and thus NADES increases, the denser crosslinked polymer network has a stronger structure, which can contain water molecules for longer periods of time. Simultaneously, since NADES is a mixture of hydrogen bond donors and acceptors, extensive hydrogen bonding takes place between the components of the hydrogels and the water molecules, and so the removal of water from the polymer matrix becomes more difficult. Moreover, from Figure 6a, it can be deduced that silver nitrate does not significantly influence retention behavior, since in most cases, a higher response (above 80%) is achieved. On the contrary, as indicated in Figure 6b, the stirring time of the sample has a major impact on the retention. As Porter et al. observed, AgNPs can act as additional anionic crosslinking sites (–COO^−^) that alter the surface charge environment of the hydrogel network, thereby restricting water uptake. Moreover, in the study by Solaiman et al., higher AgNP loadings lead to a stronger and more tightly bound polymer network, which supports the notion that, beyond a certain threshold, the presence of AgNPs reinforces the hydrogel structure and slightly affects wettability and water retention time [9,10].

#### 3.3.3. Optimization

The optimization objective was to maximize both the swelling capacity and water retention performance of the hydrogels. Based on the response surface analysis, the optimal formulation within the experimental design space was predicted to be 5 mM AgNO_3_, 4% *v*/*v* NADES-OLE, and a stirring time of 4.5 h. To validate the predictive model, two independent experiments were performed under these optimal conditions. The experimental results are summarized in Table 5. Since the mean values of the measured responses fall within the 95% prediction intervals (PIs) generated by the model, the proposed optimization conditions are considered statistically verified.

### 3.4. Nanoparticle Tracking Analysis (NTA)

**Figure 7:**
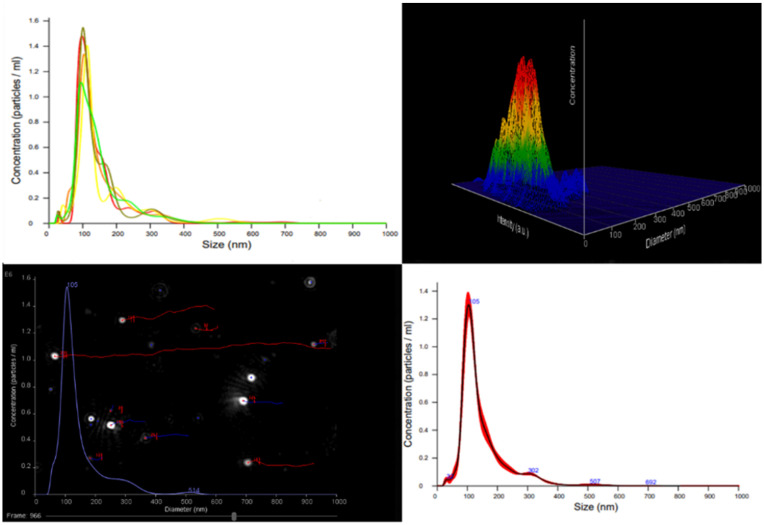
Size and concentration distribution plots for silver nanoparticles released from the nanocomposite hydrogel prepared using NADES-OLE. Different colored lines represent the concentration profiles of individual tracked nanoparticles obtained from nanoparticle tracking analysis (NTA).

The Nanoparticle Tracking Analysis (NTA) of AgNPs released from the hydrogel provided precise measurements of particle size distribution, concentration, and visualization of individual nanoparticle movement under Brownian motion in solution (Figure 7).

The released silver nanoparticles (AgNPs) exhibited an average hydrodynamic diameter of 103.2 ± 5.6 nm, with a particle concentration of (1.2 ∗ 10^8^ ± 2.2 ∗ 10^7^) particles/mL. The relatively narrow size distribution indicates that the nanocomposite hydrogel matrix effectively stabilized the AgNPs, minimizing aggregation during both synthesis and release. The measured particle size falls within the typical nanoscale range for silver nanoparticles (50–150 nm), which is suitable for biomedical and antibacterial applications. Analogous observations have been reported for AgNPs synthesized via green or Natural Deep Eutectic Solvent (NADES)-assisted approaches, where bio-based components and NADES contribute to improved nanoparticle stability and precise size control [31,32,33,34].

### 3.5. Electrophoretic Light Scattering (ELS) 

The ζ-potential of the released AgNPs was measured using ELS to assess their colloidal stability and behavior. In the prepared nanocomposite hydrogels, the nanoparticles exhibited a negative ζ-potential of −38.7 ± 1.3 mV. Values above ±30 mV are typically associated with electrostatically stable colloidal systems, indicating strong repulsive forces that prevent particle–particle collision and aggregation. Therefore, the AgNPs are effectively stabilized within the hydrogel matrix and display a minimal tendency to agglomerate.

The high negative surface charge can be attributed both to the carboxylate groups of alginate and to polyphenolic and organic acid components of the NADES-OLE system, which are known to provide steric and electrostatic stabilization via surface adsorption. According to Raus et al., alginate matrices support nanoparticle dispersion due to their anionic polysaccharide structure and abundant functional groups capable of coordinating metal ions and interacting with nanoparticle surfaces. Likewise, Wang et al. demonstrated that plant-derived polyphenols can act as natural capping and stabilizing agents for silver nanostructures, enhancing colloidal stability and preventing oxidation and aggregation [35].

Taken together, these findings suggest that the NADES–OLE system, in combination with alginate, provides a strong electrostatic and steric stabilization effect on the AgNPs, supporting long-term dispersion stability. Such stability is critical not only for preserving the structural integrity of the hydrogel but also for ensuring controlled release and sustained antibacterial activity in biomedical applications.

### 3.6. Comparative Swelling Ratio and Water Retention Ratio of the Hydrogels

**Figure 8:**
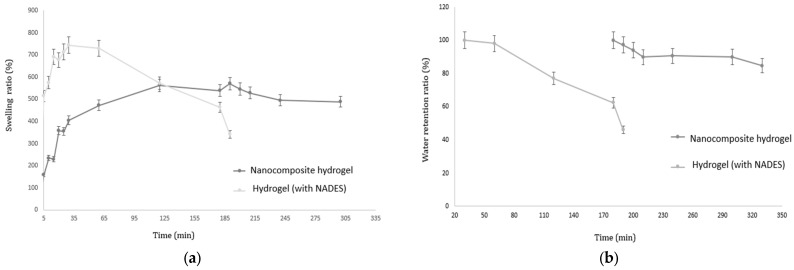
Comparative diagrams illustrating the (**a**) swelling ratio (%) in phosphate-buffered saline (PBS) solution at pH 5.5 and 37 °C and (**b**) water retention ratio (%) of nanocomposite hydrogels at pH 5.5 and a temperature of 37 °C.

The nanocomposite hydrogel containing AgNPs was compared with a hydrogel crosslinked with NADES but without AgNPs regarding swelling capacity in a phosphate-buffered saline (PBS) solution at pH 5.5 and 37 °C as well as the water retention capacity. This comparison allows the evaluation of how the incorporation of nanostructures and the NADES crosslinking mechanism influence the swelling and water retention behavior of the hydrogels (Figure 8).

The swelling behavior of the nanocomposite hydrogel, which swelled by 233% in the first 10 min and gradually increased to 538% at 180 min, suggests a controlled and sustained swelling process compared to the more rapid and higher swelling observed in the hydrogel formulation without AgNPs. This gradual swelling indicates a slower diffusion of water into the hydrogel matrix, likely due to the presence of AgNPs and NADES-OLE, which probably strengthen the hydrogel network.

The retention of 90% water at 250 min demonstrates the nanocomposite hydrogel’s ability to maintain its structure and hold moisture over an extended period. The NADES-OLE component, rich in polyphenolic compounds, likely contributes to this stability by forming strong hydrogen bonds with the sodium alginate matrix, enhancing water retention.

The role of AgNPs might also be significant in improving the hydrogel’s mechanical properties, as they can act as crosslinking agents, increasing network density. This higher density may reduce the rate of water loss, contributing to the high water retention ratio observed. The ability of this hydrogel to swell and retain water for a prolonged duration suggests its potential suitability for applications in wound dressings, drug delivery, or tissue engineering, where sustained moisture and structural integrity are crucial [24].

### 3.7. Transmission Electron Microscopy (TEM)—Energy-Dispersive Spectroscopy (EDS)

**Figure 9:**
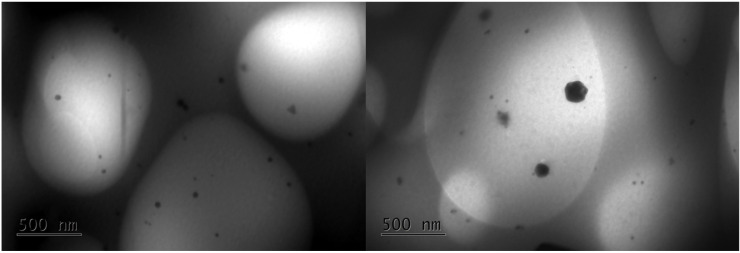
TEM images of the nanocomposite hydrogel.

Transmission electron microscopy (TEM) analysis was performed to determine the morphology and distribution of AgNPs in the polymer matrix. TEM images of the nanocomposite hydrogel (5 mM AgNO_3_, 5.4% *v*/*v* OLE, 2 h) are shown in Figure 9 and confirm that the nanoparticles are well distributed across the polymeric network and present various shapes. The smaller particles are spherical, and the larger ones have anisotropic shapes. The sizes range from 10 to 20 nm. Similar observations were also reported by Sreelekha et al., who prepared silver nanoparticles using aqueous extracts of the plant *M. frondosa* as a reducing agent [36]. The presence of only a few aggregates of AgNPs within the hydrogel network can be attributed to the ability of NADES-OLE and alginate to act as capping agents of the nanoparticles, enhancing their stability [13].

The hydrodynamic diameter measured by dynamic light scattering (DLS) was 103 nm, which is significantly larger than the 10–20 nm core sizes observed by TEM—a difference expected because DLS measures the hydrodynamic size, which includes the core particle plus its solvation (hydration) layer, any adsorbed molecules or stabilizing shell, and possible small aggregates, whereas TEM measures only the core particle diameter in the dry state [36].

**Figure 10:**
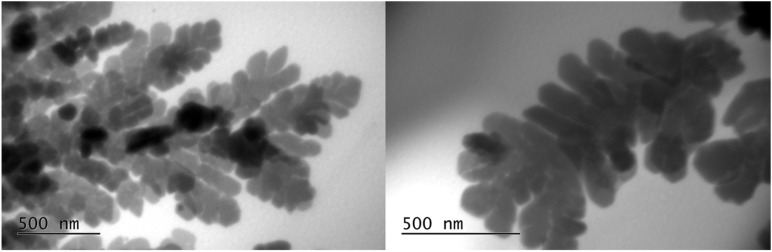
TEM images of larger silver nanostructures within the nanocomposite hydrogel.

In a few areas, some larger dendritic silver nanostructures (500 nm) were observed (Figure 10). The formation of larger growths, usually observed in the early stages of AgNP nucleation and growth, is also reported in other published studies [37,38].

**Figure 11:**
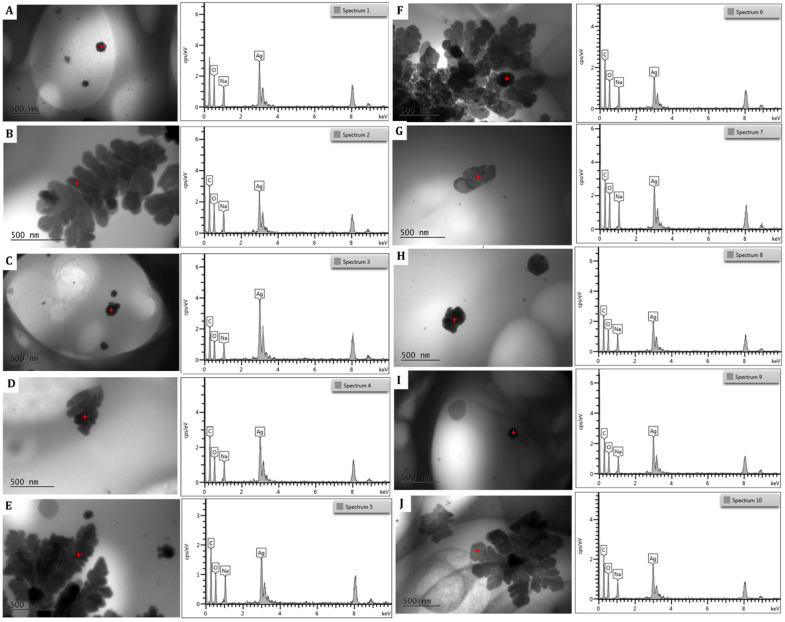
EDS spectra of the silver nanoparticles within the nanocomposite hydrogel. Letters (**A**–**J**) correspond to different EDS spectra obtained from distinct regions of the same sample, illustrating the elemental distribution and confirming the presence of silver. Red star highlights a representative nanoparticle (or cluster) for morphology characterization.

The EDS spectra of the silver nanoparticles within the hydrogel (Figure 11) showed an intense optical absorption band peak at 3 KeV. This is characteristic of the absorption of metallic AgNPs due to surface plasmon resonance. Peaks attributable to the presence of oxygen, carbon, and sodium appear due to the presence of the hydrophilic sodium alginate colloidal matrix and the NADES-OLE, which both contribute as capping agents on the surface of the AgNPs.

In the semi-quantitative analysis, silver has the highest percentage, confirming that the NPs are completely constituted by silver atoms (Table 6). The results are in agreement with those reported by Calderon-Jimenez et al. and Jebril et al. for AgNPs prepared using NaBH_4_ as a reducing agent and sonication [39,40].

### 3.8. Antibacterial Activity 

**Figure 12:**
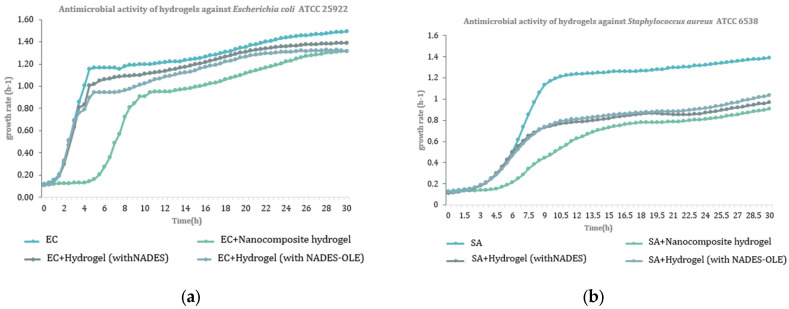
Growth curves of (**a**) Gram (−) bacterium *Escherichia coli* ATCC 25922 (EC) and (**b**) Gram (+) bacterium *Staphylococcus aureus* ATCC 6538 (SA) in the presence of the nanocomposite hydrogel (1 mg/mL), hydrogel with NADES-OLE (1 mg/mL), and the hydrogel with NADES (1 mg/mL).

The findings from the antibacterial assays for the nanocomposite hydrogels, as well as for the corresponding hydrogels without silver nanoparticles—specifically hydrogel (with NADES) and hydrogel (with NADES-OLE)—are presented in Figure 12 and in Table 7 and Table 8.

According to the results of the antibacterial activity assay, the nanocomposite hydrogel showed, at a concentration of 1 mg/mL, significant activity against the growth of Gram (−) bacteria *Escherichia coli* ATCC 25922 (64.9%) and *Yersinia enterocolitica* NCTC 12982/ATCC 9610 (60.6%), as well as against the Gram (+) bacteria *Staphylococcus aureus* ATCC 6538 (79.1%) and *Bacillus cereus* NCTC 10320/ATCC 9634 (55.3%). The hydrogel with NADES-OLE demonstrated satisfactory inhibitory activity against *Escherichia coli* ATCC 25922 (57.7%) at a concentration of 1 mg/mL. Finally, it is worth mentioning that hydrogels with NADES also showed antibacterial activity, confirming that NADES enhanced the bioactivity of the antibacterial system.

Similar studies have shown that both Gram-negative and Gram-positive bacteria could be inhibited by lactic acid-based NADES due to increased acidity that disrupts the bacterial cell membrane and impairs bacterial metabolic functions [41]. When lactic acid-based NADESs are combined with olive leaf extract, the antibacterial potency could be further enhanced through the presence of phenolic compounds such as oleuropein and hydroxytyrosol, which contribute to oxidative stress induction, cell wall destabilization, and enzyme inhibition [42,43]. Additionally, nanocomposite hydrogels containing silver nanoparticles (AgNPs) demonstrate broad-spectrum antibacterial activity, as AgNPs interact with bacterial membranes, generate reactive oxygen species, and disrupt DNA replication, while the hydrogel matrix enables controlled release and sustained contact with microbial cells [8,10].

## 4. Conclusions

In the present study, nanocomposite alginate hydrogels incorporating silver nanoparticles (AgNPs) were synthesized using a sustainable, green approach. A task-specific Natural Deep Eutectic Solvent (NADES), composed of glucose, lactic acid, and water, was utilized to extract bioactive compounds from olive leaves. This extract served two key functions: (a) as a crosslinking agent for the preparation of alginate hydrogels and (b) as a reducing agent for the in situ synthesis of AgNPs during hydrogel formation under solar light. The resulting nanocomposite hydrogels were characterized for their swelling capacity and water retention in phosphate buffer (pH 5.5), achieving 538% swelling within 180 min and 90% water retention after 250 min. Transmission electron microscopy (TEM) revealed the AgNPs had diverse morphologies, including triangular, hexagonal, and spherical shapes, with sizes ranging from 10 to 20 nm. Antibacterial testing against foodborne pathogens, including Gram-negative (*Escherichia coli*, *Yersinia enterocolitica*) and Gram-positive bacteria (*Staphylococcus aureus*, *Bacillus cereus*), demonstrated significant antibacterial activity. At a concentration of 1 mg/mL, the nanocomposite hydrogel exhibited inhibition rates of 64.9% against *E. coli*, 60.6% against *Y. enterocolitica*, 79.1% against *S. aureus*, and 55.3% against *B. cereus*, highlighting its potential for applications in antibacterial treatments.

## Figures and Tables

**Table 1:** Independent variables and responses for the Box–Behnken experimental design.

Independent Variables	Level		
Factors	Low (−1)	Medium (0)	High (1)
A: [AgNO_3_] (mM)	1	3	5
B: NADES-OLE (% *v*/*v*)	4	5	6
C: Time (Hours)	2	4	6
Responses			
R1	Swelling Ratio (SR) (%)		
R2	Water Retention Ratio (WRR) (%)		

**Table 2:** The experimental runs and observed responses for all fifteen hydrogels.

	Independent Factors						Responses	
Run	A: [AgNO_3_]		B: NADES-OLE		C: Time			
							Swelling Ratio (SR) (10 min)	Water Retention Ratio (WRR) (300 min)
	mM		% *v*/*v*		h		%	%
1	5	+1	5	0	2	−1	113	97
2	3	0	4	−1	2	−1	243	86
3	1	−1	5	0	2	−1	137	98
4	3	0	6	+1	2	−1	240	74
5	3	0	5	0	4	0	244	90
6	1	−1	6	+1	4	0	233	86
7	5	+1	6	+1	4	0	173	90
8	3	0	5	0	4	0	178	90
9	5	+1	4	−1	4	0	300	84
10	3	0	5	0	4	0	123	94
11	1	−1	4	−1	4	0	416	58
12	3	0	6	+1	6	+1	161	86
13	1	−1	5	0	6	+1	174	79
14	3	0	4	−1	6	+1	862	35
15	5	+1	5	0	6	+1	311	56

**Table 3:** Statistical significance of each model term for the swelling ratio.

	Model	Lack-of-Fit	*A*	*B*	*C*	*AB*	*AC*	*BC*	*A^2^*	*B^2^*	*C^2^*
*p*-value	0.0473	0.2175	0.8283	0.0150	0.0396	0.7905	0.4504	0.0167	0.4042	0.0362	0.840
F-value	4.91	3.75	0.0522	13.19	7.65	0.0786	0.6696	12.49	0.8295	8.07	0.9096

**Table 4:** Statistical significance of each model term for the water retention ratio.

	Model	Lack-of-Fit	*A*	*B*	*C*	*AB*	*BC*	*B* *^2^*	*C* *^2^*	*AB* *^2^*
*p*-value	0.0015	0.1091	0.0770	0.0037	0.0008	0.0985	0.0014	0.0062	0.0211	0.0146
F-value	16.43	8.41	4.55	21.03	38.67	3.82	31.32	17.05	9.62	11.51

**Table 5:** Validation of the model for a 95% confidence interval.

Responses	Predicted Mean	Predicted Median	Std Dev	n	SE Pred	95% PI Low	Data Mean	95% PI High
Water Swelling	464.23	464.23	98.80	3	111.48	177.67	437.50	750.79
Water Retention	88.88	88.88	5.36	3	6.17	73.78	87.00	103.98

**Table 6:** Semiquantitative elemental analysis οf different regions (A–J) of the nanocomposite hydrogel.

Element	Wt%									
	A	B	C	D	E	F	G	H	I	J
C	-	27.93	16.23	26.07	28.37	32.67	26.35	27.75	23.07	29.37
O	16.80	13.69	8.04	10.90	11.99	15.22	15.63	13.92	12.05	14.05
Na	8.72	7.51	4.37	5.71	7.29	7.31	8.07	7.28	5.87	6.55
Ag	74.47	50.87	71.36	57.32	52.36	44.79	49.95	51.05	59.01	50.03
Total	100.00	100.00	100.00	100.00	100.00	100.00	100.00	100.00	100.00	100.00

**Table 7:** Gram-negative bacteria growth rate (h^−1^).

	Growth Rate (h^−1^) *Escherichia coli*	Inhibition (%)	Growth Rate (h^−1^) *Yersinia enterocolitica*	Inhibition (%)
Microorganism only (no hydrogel)	0.193 ± 0.030	-	0.093 ± 0.010	-
Hydrogel (with NADES)	0.165 ± 0.010	14.2	0.080 ± 0.000	13.9
Hydrogel (with NADES-OLE)	0.082 ± 0.030	57.7	0.063 ± 0.010	31.9
Nanocomposite hydrogel	0.068 ± 0.010	64.9	0.037 ± 0.010	60.6

**Table 8:** Gram-positive microorganism growth rate (h^−1^).

	Growth Rate (h^−1^) *Staphylococcus aureus*	Inhibition (%)	Growth Rate (h^−1^) *Listeria monocytogenes*	Inhibition (%)	Growth Rate (h^−1^) *Bacillus cereus*	Inhibition (%)
Microorganism only (no hydrogel)	0.153 ± 0.035	-	0.137 ± 0.000	-	0.300 ± 0.020	-
Hydrogel (with NADES)	0.109 ± 0.007	28.7	0.102 ± 0.000	25.6	0.196 ± 0.010	34.6
Hydrogel (with NADES-OLE)	0.102 ± 0.037	33.3	0.114 ± 0.010	17.0	0.183 ± 0.030	39.0
Nanocomposite hydrogel	0.032 ± 0.009	79.1	0.102 ± 0.000	25.5	0.134 ± 0.010	55.3

## Data Availability

The original contributions presented in this study are included in the article. Further inquiries can be directed to the corresponding author.
